# An examination of pre-service teachers’ public speaking attitudes with respect to sportive activity and demographic variables

**DOI:** 10.3389/fpsyg.2025.1713142

**Published:** 2026-01-12

**Authors:** Utku Gonener, Burak Tozoglu, Fatmanur Öztürk Gonener, Besim Yildirim

**Affiliations:** 1 Faculty of Sport Sciences, Kocaeli University, Kocaeli, Türkiye; 2 Erzurum Provincial Directorate of Youth Services and Sports, Erzurum, Türkiye; 3 Faculty of Sport Sciences, Topkapı University, Istanbul, Türkiye; 4 Department of Journalism, Faculty of Communication, Atatürk University, Erzurum, Türkiye

**Keywords:** communication anxiety, physical activity, pre-service teachers, public speaking, social cognitive theory

## Abstract

**Introduction:**

Public speaking is a core professional skill for teachers, shaping classroom interaction and instructional effectiveness, yet it is frequently accompanied by anxiety and avoidance. This study examined pre-service teachers’ public speaking attitudes in relation to demographic characteristics and participation in sports activities, focusing on enjoyment, perceived importance, and anxiety.

**Method:**

A descriptive survey design was used with 464 pre-service teachers (333 female, 131 male) enrolled at the Faculty of Education of Atatürk University during the 2024–2025 academic year. Data were collected using a Personal Information Form and the Public Speaking Attitude Scale (PSAS), adapted into Turkish by Aydoğan and Çelik (2024). All sub-dimensions demonstrated strong reliability (*α* > 0.80). Independent samples *t*-tests, one-way ANOVA, and Pearson correlation analyses were conducted.

**Results:**

Male participants reported significantly higher enjoyment of public speaking, whereas female participants exhibited higher anxiety levels. Age showed a small but significant positive association with enjoyment. Family structure was significantly related to anxiety, with students from broken families reporting higher anxiety than those from nuclear or extended families. Participation in sports activities was associated with more favorable public speaking attitudes; students who engaged in sports reported greater enjoyment and lower anxiety compared to non-participants. No significant differences were observed for perceived importance.

**Conclusion:**

The findings indicate that public speaking attitudes are shaped by the combined influence of demographic and behavioral factors. In particular, sports participation appears to support the emotional dimensions of public speaking by reducing anxiety and enhancing enjoyment. These results highlight the value of integrating sport-based and socio-emotional practices into teacher education programs to strengthen communicative competence among future teachers.

## 1 Introduction

Empirical research has consistently demonstrated that public speaking proficiency is closely associated with classroom communication quality, instructional clarity, and perceived teaching effectiveness, particularly within teacher education contexts. Studies grounded in the communication apprehension framework indicate that individuals with higher oral communication competence exhibit more effective instructional performance and greater professional influence in educational settings (McCroskey, 1977; McCroskey, 1984).

However, public speaking is also recognized as a communicative challenge that provokes heightened anxiety, cognitive tension, and avoidance tendencies in many individuals. This phenomenon is conceptualized in recent literature as “communication apprehension,” encompassing physiological, emotional, and cognitive reactions triggered by the anticipation of addressing an audience. Empirical evidence further indicates that individuals with lower self-efficacy or prior negative experiences tend to develop unfavorable attitudes toward public speaking, which in turn substantially undermines their communicative performance (Schulenberg, 2023; Rahman and Pinky, 2023; Zarrinabadi and Khodarahmi, 2023).

Communication apprehension is not confined to an individual’s internal psychological state but is also shaped by social environments, learning experiences, and cognitive processes. Social cognitive perspectives emphasize the role of self-efficacy, defined as the individual’s belief in their ability to execute specific behaviors, in mitigating communication-related anxiety. Recent studies confirm that individuals with higher self-efficacy display less anxiety and communicate more effectively in socially demanding contexts, whereas low self-efficacy predicts heightened apprehension (Zarrinabadi and Khodarahmi, 2023; Schunk and DiBenedetto, 2022).

In parallel, physical and sport activities—recognized as central to psychosocial development—have been extensively examined for their impact on communication competencies. Empirical reviews demonstrate that regular physical activity reduces psychological risks such as stress, depression, and social isolation, while simultaneously fostering self-efficacy, self-esteem, and social connectedness (Reznik and Wylie-Rosett, 2022; Rodriguez-Ayllon et al., 2022). These engagements may enhance psychological resilience and protect against communication-related anxiety during public speaking tasks.

Despite the expanding body of literature, studies conducted in Turkey still rarely integrate public speaking attitudes with variables such as sport participation and socio-demographic characteristics within a unified framework. Existing research has predominantly examined communication skills, public speaking anxiety, or the psychological benefits of physical activity in isolation, rather than through a comprehensive theoretical lens. However, teaching extends beyond the mere transmission of knowledge and requires confident verbal communication, effective classroom presence, and the ability to establish meaningful interpersonal interaction with students. Prior research highlights that communication apprehension and self-perceived communicative competence play a central role in instructional effectiveness and classroom engagement (McCroskey, 1984; MacIntyre and Khajavy, 2022). Accordingly, examining pre-service teachers’ public speaking attitudes within an integrated and multidimensional framework remains essential for advancing both individual professional development and teacher education practices. Accordingly, this study aims to examine the public speaking attitudes of pre-service teachers in relation to variables such as gender, age, family structure, level of education, sports participation, and type of preferred sport. The study further seeks to identify whether sports participation plays a predictive role in shaping these attitudes. The findings are expected to provide new insights for improving pre-service teachers’ communicative competencies, and to contribute to building an interdisciplinary bridge between communication sciences and sports sciences by revealing how physical activity may impact social-cognitive structures. In this respect, the study offers a unique perspective that contributes both theoretically and practically to the teacher education literature. From a social cognitive perspective, the relationship between sports participation and public speaking attitudes can be explained through self-efficacy–based mechanisms. Social Cognitive Theory posits that individuals develop beliefs about their capabilities through mastery experiences, social modeling, and social persuasion, all of which are commonly embedded in sports participation contexts. Regular engagement in sports provides repeated performance experiences, opportunities for feedback, and exposure to evaluative social situations, which may strengthen individuals’ confidence in managing performance-related stress and audience-focused tasks.

In this framework, self-efficacy is assumed to play a mediating role between sports participation and public speaking attitudes. Individuals who perceive themselves as competent in physical and social performance settings may generalize this confidence to other evaluative contexts, such as public speaking. Higher self-efficacy may therefore be associated with greater enjoyment and lower anxiety during public speaking, not because sports participation directly causes these outcomes, but because it is linked to psychological resources that support adaptive communication-related beliefs and emotional regulation.

## 2 Methods

This study utilized a descriptive survey design—a quantitative approach that aims to systematically profile a phenomenon, individual, or context under existing conditions without researcher intervention. Contemporary research methodology underscores the utility of descriptive surveys for capturing real- world patterns and behavioral trends (Johnson and Christensen, 2023; Creswell and Creswell, 2023). Within this framework, a relational or correlational survey design was adopted, facilitating statistical assessment of relationships among variables and enabling insights into potential predictive links (Saunders et al., 2022; Cohen et al., 2021).

In the present research, variables were not manipulated; instead, naturally occurring relationships were observed and statistically tested. Accordingly, the study is structured to explore how demographic and behavioral factors—especially engagement in sports activities—relate to pre-service teachers’ attitudes toward public speaking, preserving ecological validity while investigating meaningful associations.

Data were analyzed using SPSS version 26.0. Prior to the analyses, normality assumptions were examined through skewness and kurtosis values, which were found to be within acceptable ranges. Independent samples *t*-tests were conducted to examine differences based on gender and sports participation, while one-way ANOVA was used to compare public speaking attitudes across family structure, grade level, and type of sports activity. Pearson correlation analysis was performed to examine relationships between age, weekly duration of sports activity, and public speaking attitudes. Statistical significance was set at *p* < 0.05.

### 2.1 Population and sample

The population of this study consisted of students enrolled in the Kazım Karabekir Faculty of Education, Atatürk University, during the 2024–2025 academic year. The final sample comprised 464 voluntary participants (333 female and 131 male). A convenience sampling method was employed, a widely used non-probability approach in quantitative research due to its practicality and efficiency in data collection (Robinson, 2023; Etikan, 2022). This technique involves selecting participants who are readily accessible and willing to take part in the study, offering logistical advantages under time and resource constraints (Daniel, 2022).

Despite these advantages, convenience sampling has inherent limitations regarding representativeness.

Since the method does not guarantee proportional coverage of the population, it may reduce internal and external validity, especially for studies seeking statistical generalization (Acharya and Sharma, 2023). Furthermore, the voluntary nature of participation introduces the risk of self-selection bias, as individuals with specific interests—such as stronger motivation toward public speaking or sports participation—may be overrepresented (Robinson, 2023).

Therefore, caution is warranted when generalizing the findings beyond the studied population. Future research may enhance representativeness by employing probability-based sampling strategies, such as stratified or multistage cluster sampling, which are more effective in ensuring subgroup balance and improving the robustness of statistical inference (Saunders et al., 2022; Daniel, 2022).

### 2.2 Data collection instruments and procedures

Data were collected during the spring semester of the 2024–2025 academic year through fieldwork conducted face-to-face with pre-service teachers enrolled in undergraduate programs at the Kazım Karabekir Faculty of Education, Atatürk University. The data collection process spanned approximately 6 weeks (April–May 2025) and was carried out during class sessions, avoiding examination periods to minimize academic burden. All participants were informed verbally and in writing about the study’s aims, confidentiality, and voluntary participation.

The data collection instrument consisted of two sections. The first section included a Personal Information Form designed to collect demographic information such as gender, age, family structure, year of study, and participation in physical activity. The second section focused on attitudes toward public speaking, a construct closely associated with communication apprehension and affective responses to oral communication situations. Public speaking attitudes have been conceptualized in the literature as a multidimensional phenomenon encompassing affective reactions, perceived competence, and anxiety related to evaluative speaking contexts (McCroskey, 1977; McCroskey and Richmond, 1982). Previous research has demonstrated that anxiety and negative affect toward public speaking are systematically related to individuals’ communication experiences and behavioral engagement in speaking situations (Allen et al., 1989). Accordingly, public speaking attitudes were assessed using a Likert-type format, with higher scores indicating more positive evaluations and lower levels of apprehension toward public speaking tasks.

Structural validity of the scale was tested using Confirmatory Factor Analysis (CFA) with AMOS 24.0. Prior to CFA, assumptions of multivariate analysis were examined. Multivariate normality was evaluated through Mardia’s coefficient and standardized z-values, and Mahalanobis distances were calculated to detect multivariate outliers (Byrne, 2021; Hair et al., 2022). Adequacy of sample size was confirmed based on recommendations of at least 10 observations per estimated parameter (Brown, 2015). Multicollinearity diagnostics using Variance Inflation Factor (VIF) and tolerance values indicated no collinearity issues (Kline, 2023).

The CFA yielded acceptable fit indices: χ^2^/df = 2.84, RMSEA = 0.063, SRMR = 0.047, CFI = 0.957, TLI = 0.945, and GFI = 0.927. These values satisfy conventional thresholds (RMSEA and SRMR < 0.08; CFI and TLI > 0.90), supporting the three-factor model (Hu and Bentler, 2020; Schermelleh-Engel et al., 2003). Factor loadings ranged between 0.62 and 0.88, and all items significantly loaded onto their respective latent constructs. These findings confirm that the PSAS demonstrates robust factorial validity and can be reliably applied in the present study.

### 2.3 Ethical approval and informed consent

The study protocol was reviewed and approved by the Atatürk University Social and Humanities Ethics Committee (Decision No: 109, Document No: E.88656144-000-2500121884, April 14, 2025). All procedures complied with the ethical standards of the Declaration of Helsinki and the Council of Higher Education in Türkiye. Written informed consent was obtained from all participants, and anonymity and confidentiality were strictly maintained.

### 2.4 Reliability of the study

In this study, internal consistency reliability of the sub-dimensions of the Public Speaking Attitude Scale was examined using Cronbach’s alpha coefficients. Cronbach’s alpha is a widely used indicator of internal consistency, reflecting the extent to which items within a scale measure the same underlying construct (Cronbach, 1951). Following classical psychometric guidelines, alpha values around 0.70 or higher are generally considered acceptable for research purposes, particularly in the social sciences (Nunnally and Bernstein, 1994). However, contemporary methodological literature emphasizes that alpha values should be interpreted with caution and in relation to the number of items and the conceptual coherence of the scale rather than rigid cutoff points (Tavakol and Dennick, 2011). Accordingly, the reliability coefficients for the Enjoyment, Importance, and Anxiety sub-dimensions are reported in Table 1 to provide transparent evidence of the internal consistency of the scale within the present sample.

**Table 1:** Reliability coefficients (Cronbach’s alpha) for the sub-dimensions of the Public Speaking Attitude Scale.

Sub-dimension	Cronbach’s alpha	*N* of items
Enjoyment	0.93	8
Importance	0.87	5
Anxiety	0.87	3

In this context, the results showed that all sub-dimensions of the scale demonstrated Cronbach’s Alpha values above 0.80, indicating a high level of internal consistency. These findings suggest that the measurement tool used in the study is highly reliable and appropriate for the current sample.

According to Table 1, the reliability coefficients for the Public Speaking Attitude Scale were found to be high across all sub-dimensions. Specifically, the *Enjoyment* sub-dimension had a Cronbach’s alpha of 0.93, the *Importance* sub-dimension had 0.87, and the *Anxiety* sub-dimension had 0.87. Since all values exceed the 0.80 threshold, the scale demonstrates strong internal consistency. These findings confirm that the scale is a reliable tool for measuring pre-service teachers’ attitudes toward public speaking in the current sample.

## 3 Results

This section presents the results of the study conducted to examine pre-service teachers’ public speaking attitudes in relation to demographic variables and participation in sports activities. The findings are reported in a structured manner, beginning with reliability and descriptive statistics, followed by comparisons across demographic characteristics and sports participation variables, and concluding with correlation analyses.

According to Table 2, the descriptive statistics for the sub-dimensions of the Public Speaking Attitude Scale indicate that the data are normally distributed. Skewness and kurtosis values for Enjoyment, Importance, and Anxiety all remained within the acceptable ±1.50 range, confirming the suitability of the dataset for parametric analyses. The mean scores were 24.08 for Enjoyment, 20.19 for Importance, and 8.62 for Anxiety, reflecting moderate enjoyment, perceived importance, and anxiety levels among participants.

**Table 2:** Descriptive statistics of the sub-dimensions of the Public Speaking Attitude Scale.

Statistic	Enjoyment	Importance	Anxiety
*N*	464	464	464
Mean	24.08	20.19	8.62
Median	24.00	21.00	9.00
Mode	24.00	25.00	9.00
Std. Deviation	7.26	4.32	3.20
Skewness	−0.014	−1.146	0.175
Std. Error of Skewness	0.113	0.113	0.113
Kurtosis	−0.354	1.274	−0.488
Std. Error of Kurtosis	0.226	0.226	0.226

According to Table 3, a total of 464 pre-service teachers participated in the study, of whom 333 were female and 131 were male. Regarding family structure, 389 participants reported being raised in a nuclear family, 60 in an extended family, and 15 in a broken or single-parent family. The distribution of participants across grade levels appears to be relatively balanced, which contributes to the homogeneity of the sample and supports the statistical reliability of the analyses conducted.

**Table 3:** Distribution of participants’ demographic characteristics.

Variable	Category	*N*	%	Cumulative %
Gender	Female	333	71.8	71.8
	Male	131	28.2	100.0
	Total	464	100.0	—
Family structure	Nuclear Family	389	83.8	83.8
	Extended Family	60	12.9	96.8
	Divorced/Separated	15	3.2	100.0
Grade level	First Year	48	10.3	10.3
	Second Year	138	29.7	40.0
	Third Year	187	40.3	80.4
	Fourth Year	91	19.6	100.0

According to Table 4, the average age of participants was 22.02, with both the median and mode values recorded as 21.00. The standard deviation of 3.13 indicates a relatively homogeneous distribution, suggesting that the sample primarily consisted of individuals within a similar developmental stage.

**Table 4:** Mean age of participants.

*N*	464
Mean	22.02
Median	21.00
Mode	21.00
Std. Deviation	3.13

According to Table 5, out of the total sample of 464 individuals, 212 (45.7%) regularly participated in sports activities, while 252 (54.3%) reported no involvement. Among those who engaged in sports, 138 (29.7%) were involved in individual sports, 33 (7.1%) in team sports, and 41 (8.8%) in both individual and team sports. This distribution highlights a notable diversity in participants’ preferences, with individual sports being the most commonly chosen type of activity among pre-service teachers.

**Table 5:** Distribution of participants by status of engaging in sports activities.

Variable	Category	*N*	%	Cumulative %
Engagement in sports activity	Yes	212	45.7	45.7
	No	252	54.3	100.0
	Total	464	100.0	—
Type of sports activity	Individual Sports	138	29.7	29.7
	Team Sports	33	7.1	36.9
	Both	41	8.8	45.7
	None	252	54.3	100.0

According to Table 6, the average weekly duration of physical activity among the 212 participants who reported engaging in sports was 5.48 h (SD = 4.23). The median value was 5 h, while the modewas 2 h, indicating that the majority of participants exercised approximately 2 h per week. However, the higher mean value suggests that some participants engaged in considerably longer durations of activity, creating a positively skewed distribution. Out of the total sample of 464 individuals, 252 did not provide valid responses to this question, and therefore, statistical calculations were conducted only on the 212 valid cases.

**Table 6:** Average weekly duration of participants’ physical activity engagement.

Statistic		
*N*	Valid	212
	Invalid	252
	Mean	5.48
	Median	5.00
	Mode	2.00
	Std. Deviation	4.23

According to Table 7, the independent samples *t*-test results revealed statistically significant differences in the Enjoyment (*p* = 0.003) and Anxiety (*p* = 0.031) sub-dimensions of the Public Speaking Attitude Scale between male and female participants. Male students (*M* = 25.65, SD = 7.17) reported significantly higher levels of enjoyment compared to female students (*M* = 23.46, SD = 7.24). Conversely, female students (*M* = 8.82, SD = 3.15) exhibited significantly higher levels of anxiety than male students (*M* = 8.11, SD = 3.31). These findings suggest that male students approach public speaking with greater confidence and enjoyment, while female students are more likely to experience communicative anxiety. However, no significant gender difference was found in the Importance sub-dimension (*p* = 0.055), indicating similar perceptions of the importance of public speaking across both groups. Male students reported significantly higher enjoyment than female students (*p* = 0.003), with a small-to-moderate effect size (Cohen’s d = 0.30). Female students exhibited higher anxiety levels than males (*p* = 0.031), corresponding to a small effect size (Cohen’s d = 0.22).

**Table 7:** Comparison of participants’ public speaking attitude levels by gender.

Dimension	Gender	*N*	Mean	SD	*t*	*p*
Enjoyment	Female	333	23.46	7.24	−2.948	0.003
	Male	131	25.65	7.17		
Importance	Female	333	20.45	4.08	2.080	0.055
	Male	131	19.53	4.84		
Anxiety	Female	333	8.82	3.15	2.166	0.031
	Male	131	8.11	3.31		

According to Table 8, the correlation analysis indicated a positive and statistically significant relationship between age and the Enjoyment sub-dimension (r = 0.137, p = 0.003). This suggests that as participants’ age increases, they are more likely to develop positive and confident attitudes toward public speaking. In contrast, no significant relationships were observed between age and the Importance (r = −0.001, *p* = 0.989) or Anxiety (r = −0.066, *p* = 0.157) sub-dimensions. These results demonstrate that while age contributes to higher enjoyment of public speaking, it does not significantly influence participants’ perceptions of importance or their levels of anxiety in communicative situations.

**Table 8:** Correlation analysis results between participants’ ages and their public speaking attitudes.

Variable	Statistic	Enjoyment	Importance	Anxiety
Age	*r*	0.137	−0.001	−0.066
	*p*	0.003	0.989	0.157

According to Table 9, the one-way ANOVA results revealed a statistically significant difference in the Anxiety sub-dimension of the Public Speaking Attitude Scale based on family structure (*F* = 8.321, *p* < 0.001). *Post-hoc* comparisons indicated that participants from fragmented families reported significantly higher anxiety levels compared to those from nuclear and extended families (*p* = 0.008). This suggests that family structure may play an influential role in shaping individuals’ communicative confidence, with fragmented family backgrounds being associated with heightened public speaking anxiety.

**Table 9:** Comparison of participants’ public speaking attitudes according to family structure.

Dimensions	Family structure	*N*	x̅	SD	ANOVA		
					*f*	*p*	Difference
Enjoyment	Core	389	24.18	7.18	2.239	0.108	
	Wide	60	24.37	7.66			
	Fragmented	15	20.20	7.17			
	Total	464	24.08	7.26			
Importance	Core	389	20.19	4.28	0.019	0.981	
	Wide	60	20.17	4.77			
	Fragmented	15	20.40	3.81			
	Total	464	20.19	4.32			
Anxiety	Core	389	8.44	3.13	8.321	0.000 0.008	1 < 3 2 < 3
	Wide	60	8.98	3.18			
	Fragmented	15	11.73	3.75			
	Total	464	8.62	3.20			

In contrast, no significant differences were observed across family structures in the Enjoyment (*F* = 2.239, *p* = 0.108) and Importance (*F* = 0.019, *p* = 0.981) sub-dimensions. These findings imply that while family structure appears to be a determinant for anxiety, it does not substantially affect the enjoyment or perceived importance of public speaking.

According to Table 10, the results of the one-way ANOVA showed no statistically significant differences in public speaking attitudes across grade levels. Specifically, the Enjoyment (*F* = 0.956, *p* = 0.413), Importance (*F* = 1.535, *p* = 0.205), and Anxiety (*F* = 0.575, *p* = 0.632) sub-dimensions did not vary meaningfully among first-, second-, third-, and fourth-year students.

**Table 10:** Comparison of participants’ public speaking attitudes according to grade level.

Dimensions	Grade level	*N*	x̅	SD	ANOVA	
					*f*	*p*
Enjoyment	First Grade	48	23.88	5.72	0.956	0.413
	Second Grade	187	23.49	7.12		
	Third Grade	138	24.34	7.63		
	Fourth Grade	91	24.99	7.69		
	Total	464	24.08	7.26		
Importance	First Grade	48	20.52	4.03	1.535	0.205
	Second Grade	187	19.67	4.33		
	Third Grade	138	20.59	4.48		
	Fourth Grade	91	20.48	4.16		
	Total	464	20.19	4.32		
Anxiety	First Grade	48	9.04	2.49	0.575	0.632
	Second Grade	187	8.71	3.41		
	Third Grade	138	8.39	3.35		
	Fourth Grade	91	8.55	2.88		
	Total	464	8.62	3.20		

These findings suggest that pre-service teachers’ attitudes toward public speaking remain relatively stable throughout their undergraduate education. In other words, progression through grade levels does not appear to significantly enhance or diminish students’ enjoyment, perceived importance, or anxiety related to public speaking.

According to Table 11, independent samples *t*-test results demonstrated significant differences in the Enjoyment and Anxiety sub-dimensions with respect to engagement in sports activities. Specifically, participants who engaged in sports reported significantly higher enjoyment scores (*M* = 25.83, SD = 7.46) compared to those who did not (*M* = 22.61, SD = 6.77), *t*(462) = 4.871, *p* < 0.001. Conversely, non-participants displayed significantly higher anxiety levels (*M* = 9.08, SD = 3.16) compared to participants (*M* = 8.08, SD = 3.18), *t*(462) = −3.386, *p* = 0.001.

**Table 11:** Comparison of participants’ public speaking attitude levels based on their engagement in sportive activities.

Dimensions	Sports activity status	*N*	x̅	SD	*T* test	
					*t*	*p*
Enjoyment	Yes	212	25.83	7.46	4.871	0.000
	No	252	22.61	6.77		
Importance	Yes	212	20.40	4.20	0.966	0.335
	No	252	20.01	4.42		
Anxiety	Yes	212	8.08	3.18	−3.386	0.001
	No	252	9.08	3.16		

No significant difference was observed in the Importance sub-dimension (*p* = 0.335), indicating that participation in physical activity primarily influences emotional aspects of public speaking (i.e., enjoyment and anxiety), rather than perceptions of its importance. These findings suggest that sports involvement contributes to reducing communication-related anxiety and enhancing enjoyment in public speaking contexts, thereby indirectly supporting the development of communicative competence among pre-service teachers.

Participants who engaged in sports reported higher enjoyment (*p* < 0.001), with a moderate effect size (Cohen’s d = 0.45), and lower anxiety (*p* = 0.001), reflecting a small-to-moderate effect size (Cohen’s d = 0.32) compared to non-participants.

According to Table 12, one-way ANOVA results demonstrated significant differences in the Enjoyment and Anxiety sub-dimensions across different types of sports participation. In the Enjoyment sub-dimension, individuals engaged in individual sports (*M* = 25.83, SD = 7.55) and team sports (*M* = 26.40, SD = 7.53) reported significantly higher scores compared to those who did not participate in any sports (*M* = 22.61, SD = 6.77), *F*(3, 460) = 8.010, *p* < 0.001. *Post-hoc* comparisons confirmed that both individual and team sport participants showed greater enjoyment than non-participants (*p* = 0.000, *p* = 0.022).

**Table 12:** Comparison of participants’ public speaking attitude levels according to type of sports activity.

Dimensions	Type of sports activity	*N*	x̅	SD	ANOVA		
					*f*	*p*	Difference
Enjoyment	Individual Sports	138	25.83	7.55	8.010	0.000 0.022	1 > 4 2 > 4
	Team Sports	33	26.40	7.53			
	Both	41	25.37	7.23			
	I Do not	252	22.61	6.77			
	Total	464	24.08	7.26			
Importance	Individual Sports	138	20.70	4.02	0.967	0.488	
	Team Sports	33	19.73	4.10			
	Both	41	19.93	4.82			
	I Do not	252	20.01	4.42			
	Total	464	20.19	4.32			
Anxiety	Individual Sports	138	8.33	3.36	4.769	0.010	3 < 4
	Team Sports	33	7.85	3.00			
	Both	41	7.42	2.64			
	I Do not	252	9.08	3.16			
	Total	464	8.62	3.20			

In terms of Anxiety, students who reported no sports participation exhibited significantly higher levels of anxiety (*M* = 9.08, SD = 3.16) compared to those engaged in team sports (*M* = 7.85, SD = 3.00) or both types of sports (*M* = 7.42, SD = 2.64), *F*(3, 460) = 4.769, *p* = 0.010. These results suggest that participation in sports, particularly team-based or combined activities, is associated with reduced communication-related anxiety.

No significant differences were observed in the Importance sub-dimension (*p* = 0.488), indicating that sports engagement primarily affects emotional dimensions of public speaking attitudes rather than the perceived significance of the skill. Overall, the findings reinforce the role of physical activity as a psychosocial factor that enhances enjoyment and mitigates anxiety in public speaking contexts.

As shown in Table 13, the correlation analysis results indicated no statistically significant relationship between the weekly duration of physical activity and participants’ attitudes toward public speaking.

**Table 13:** Correlation analysis results between participants’ duration of sports activities and their public speaking attitudes.

Variable		Enjoyment	Importance	Anxiety
Exercise time (Weekly Hours)	*r*	−0.109	−0.029	−0.006
	*p*	0.112	0.672	0.929

Specifically, the correlations between exercise time and the Enjoyment (*r* = −0.109, *p* = 0.112), Importance (*r* = −0.029, *p* = 0.672), and Anxiety (*r* = −0.006, *p* = 0.929) sub-dimensions were not significant. These results suggest that the amount of time spent on physical activity alone does not serve as a meaningful predictor of public speaking attitudes. Rather than duration, factors such as the type of sport, level of social interaction involved, and the individual’s intrinsic motivation toward physical activity may play a more substantial role in shaping communicative attitudes. This finding emphasizes the importance of qualitative aspects of physical activity over quantitative measures when considering its potential impact on psychological and communicative outcomes.

## 4 Discussion

This study aimed to examine whether prospective teachers’ attitudes toward public speaking differ significantly across demographic and behavioral variables. The findings indicate that public speaking attitudes are shaped by the combined influence of demographic characteristics, psychosocial context, and behavioral engagement rather than representing fixed individual traits. This pattern is consistent with research emphasizing that communication apprehension and speaking-related attitudes are context-dependent and influenced by multiple interacting factors (Zarrinabadi and Khodarahmi, 2023).

Gender differences emerged as a salient factor associated with public speaking attitudes. Male participants reported higher levels of enjoyment, whereas female participants exhibited higher anxiety scores. These findings align with prior empirical evidence showing that female students often report greater communication apprehension, particularly in evaluative or performance-oriented speaking contexts (Schulenberg, 2023; Rahman and Pinky, 2023). Previous studies suggest that gendered social expectations and perceived evaluative pressure may contribute to heightened anxiety among female speakers (Schunk and DiBenedetto, 2022). Moreover, cross-cultural research demonstrates that the expression and intensity of communication apprehension may vary according to sociocultural norms, underscoring the importance of contextual factors in interpreting gender-related differences (Zarrinabadi and Khodarahmi, 2023).

Age-related differences were also observed, with older participants reporting greater enjoyment of public speaking. This pattern suggests that accumulated experience and maturity may be associated with increased confidence in communication settings. From a social cognitive perspective, repeated mastery experiences contribute to the development of self-efficacy, which in turn supports more adaptive responses in performance-based tasks such as public speaking (Schunk and DiBenedetto, 2022). Accordingly, age-related variations in enjoyment may reflect gradual changes in self-regulatory skills and perceived competence rather than developmental causality.

Family structure was another factor associated with public speaking anxiety. Participants from disrupted family backgrounds reported higher anxiety levels compared to those from nuclear or extended families. This finding is consistent with research highlighting the protective role of social and familial support in buffering stress and anxiety (Cutrona and Russell, 1990). Supportive family environments may facilitate emotional regulation and adaptive coping strategies, which are particularly relevant in situations involving evaluative communication demands. The present results suggest that family-related psychosocial factors may continue to influence communication-related anxiety within educational and professional training contexts.

Engagement in sports activities was also associated with public speaking attitudes. Students who reported regular participation in physical activity demonstrated higher enjoyment and lower anxiety levels related to public speaking. These findings are consistent with evidence indicating that physical activity is linked to improved psychological well-being, including reduced anxiety and enhanced emotional resilience (Rodriguez-Ayllon et al., 2022). Physical activity may indirectly support communication-related outcomes by fostering self-confidence, stress regulation, and social connectedness. However, no significant association was observed between the weekly duration of physical activity and public speaking attitudes, suggesting that participation itself rather than frequency may be more relevant to these outcomes resilience.

The findings have important implications for teacher education programs. Public speaking competence is a fundamental professional skill for educators, influencing classroom management, instructional clarity, and teacher–student interaction. Integrating communication-focused training with psychosocial and physical activity–based practices may provide a more comprehensive approach to supporting prospective teachers’ communicative development. Programs that promote emotional regulation, self-efficacy, and stress management may be particularly beneficial for students who experience higher levels of communication apprehension.

Despite these contributions, several limitations should be considered. The use of convenience sampling from a single institution limits the generalizability of the findings. In addition, the cross-sectional design does not allow for causal interpretations, and the observed associations should be understood as correlational in nature. Furthermore, although sports participation was assessed, qualitative aspects such as motivation, perceived benefits, and social dynamics of engagement were not examined. Future research employing longitudinal, multi-institutional, and mixed-methods designs may provide a more comprehensive understanding of the mechanisms underlying public speaking attitudes.

In addition, several potentially confounding variables were not measured or controlled for in the present study. Factors such as prior public speaking experience, individual personality traits (e.g., extraversion), academic performance, and socioeconomic status may influence both engagement in sports activities and attitudes toward public speaking. The absence of these variables in the analyses may have contributed to the observed relationships and limits the ability to isolate the unique role of sports participation. Future research incorporating a broader set of psychological, educational, and contextual covariates would provide a more comprehensive understanding of the mechanisms underlying public speaking attitudes. In addition, information regarding response rates and non-respondents was not systematically recorded, which limits the ability to assess potential non-response bias.

In conclusion, this study contributes to the growing literature on communication in education by identifying demographic, familial, and behavioral determinants of public speaking attitudes among prospective teachers. By demonstrating that gender, age, family structure, and sports participation are significant predictors of communicative enjoyment and anxiety, the results highlight the necessity of comprehensive educational interventions. Teacher training programs that combine communication skill-building with psychosocial and physical activity-based practices may not only reduce anxiety but also foster confidence, resilience, and professional competence in future educators.

## 5 Conclusion

This study investigated pre-service teachers’ attitudes toward public speaking in relation to demographic variables, family background, and engagement in sports activities. The findings demonstrated that public speaking attitudes are shaped by a combination of individual and contextual factors, highlighting the multidimensional nature of communicative competence.

Gender and family structure emerged as critical determinants, with female students reporting higher anxiety levels and students from disrupted families experiencing greater communicative apprehension.

Age was positively associated with enjoyment in public speaking, suggesting that maturity and accumulated experience contribute to greater communicative confidence. Importantly, engagement in sports activities significantly enhanced enjoyment and reduced anxiety, while the type of sport appeared more influential than the frequency of participation.

These results underscore the importance of integrating communication training with psychosocial and sport-based practices in teacher education curricula. Public speaking is a core professional skill for educators, directly affecting classroom performance and student engagement. Therefore, interventions that combine cognitive-behavioral techniques, mentorship, and structured physical activity programs may help reduce anxiety, foster resilience, and strengthen professional identity among future teachers.

Despite its contributions, the study is limited by its cross-sectional design, reliance on self-report measures, and the use of convenience sampling within a single institution. Future research should adopt longitudinal and multi-institutional approaches, incorporate qualitative insights into motivational and cultural factors, and evaluate intervention-based programs to provide a more comprehensive understanding.

In conclusion, the findings contribute both theoretically and practically to the literature on communication and teacher education. By demonstrating the role of demographic, familial, and behavioral variables—particularly the positive impact of sports participation—the study highlights the need for holistic, interdisciplinary approaches to developing effective communication competencies in pre-service teachers.

## Data Availability

The raw data supporting the conclusions of this article will be made available by the authors, without undue reservation.
